# Supplementary material for: Vitamin D and ω-3 Supplementations in Mediterranean Diet During the 1st Year of Overt Type 1 Diabetes: A Cohort Study
Source: Nutrients. 2019 Sep 9;11(9):2158. doi: 10.3390/nu11092158 (PMC6770446; doi:10.3390/nu11092158)
Supplement: Supplementary file 1 [file nutrients-11-02158-s001.pdf]

Supplementary Materials:

A structured Dietician Training on a Mediterranean-Style Diet

The recommended composition of the dietary regimen was suggested as: 50–55% of carbohydrates, 10–15% of proteins or 0.80–1.34 g/Kg/d according to age and gender, 30–35% of total fats of which saturated less than 10%, and a cholesterol consumption of 100 mg every 1000 Kcal. Two or more servings of fish per week and two servings of fruit per day (400 g), as well as three of vegetables (300 g) per day were also recommended. Walnut and olive oil consumption was suggested. A fiber intake of 12–14 g for every 1000 Kcal, and intake of whole foods was encouraged, as was choosing a variety of fiber-rich foods (whole grains, fruits, vegetables, vegetable soups, etc.). The nutritional counseling was scheduled into three structured and managed steps.

The first step was aimed at detecting the proportion of daily caloric intake (carbohydrates, proteins, and lipids) and promoting the intake of monounsaturated fatty acids. Rates of lipids were calculated according to age. The second step was more structured, and detected the intake of fibers, fruits, and vegetables as part of the daily diet, and their consumption was increased and promoted. A gradually progressive increased consumption of fibers was suggested in order to reduce a refusal due to palatability and/or gastrointestinal side effects. The third step was aimed at giving a better explanation of the concept of serving size and lifestyle flexibility, in order to manage exceptions and fit in events that would not allow usual meal consumption. This step implied the use of pictorial copies of meals and nutrients, as well as booklets. One hour per step was reserved for every subject and/or their parents [1].

1. Cadario, F.; Prodam, F.; Pasqualicchio, S.; Bellone, S.; Bonsignori, I.; Demarchi, I.; Monzani, A.; Bona, G. Lipid profile and nutritional intake in children and adolescents with Type 1 diabetes improve after a structured dietician training to a Mediterranean-style diet. *J. Endocrinol. Investig.* **2012**, *35*, 160–168.
